# Multivariate nonparametric chart for influenza epidemic monitoring

**DOI:** 10.1038/s41598-019-53908-6

**Published:** 2019-11-25

**Authors:** Liu Liu, Jin Yue, Xin Lai, Jianping Huang, Jian Zhang

**Affiliations:** 1 School of Mathematics and V.C. & V.R. Key Lab, Sichuan Normal University, Chengdu, China; 2 School of Computer Science and Technology, Xi’an Jiaotong University, Xi’an, China; 3 School of Mathematics, Sichuan University of Arts and Science, Dazhou, China; 4 School of Geosciences, China University of Petroleum(East China), Qingdao, China; 5 School of Mathematical Sciences, University of Electronic Science and Technology of China, Chengdu, China

**Keywords:** Statistics, Epidemiology

## Abstract

Control chart methods have been received much attentions in biosurvillance studies. The correlation between charting statistics or regions could be considerably important in monitoring the states of multiple outcomes or regions. In addition, the process variable distribution is unknown in most situations. In this paper, we propose a new nonparametric strategy for multivariate process monitoring when the distribution of a process variable is unknown. We discuss the EWMA control chart based on rank methods for a multivariate process, and the approach is completely nonparametric. A simulation study demonstrates that the proposed method is efficient in detecting shifts for multivariate processes. A real Japanese influenza data example is given to illustrate the performance of the proposed method.

## Introduction

Control charts are useful tools for fault detection^1^. Shewhart chart, CUSUM chart and EWMA chart are most popular tools in statistical process control. These control charts are efficient and fruitful for fault diagnosis in practical applications. Most control charts that need observations are univariate and usually assume that the observation follows a known gaussian distribution.

In real life, we usually process multivariate or high-dimensional variables rather than univariate variables. The monitoring of high–dimensional data in a timely manner has become increasingly important in quality control. Hotelling^2^ proposed a T-squared control chart for multivariate process, which assumes that the dataset distributions are multivariate normal distribution. Both the parameters of mean vector and variance matrix are known. Based on *T*^2^ statistics, Lowry *et al*.^3^ proposed a multivariate CUSUM chart. Furthermore, Sullivan and Woodall^4^ provided a change–point chart for detecting a shift of the location parameter, the scale parameter.

However, statistical process control is a challenge when the underlying distribution and the magnitude of changes are both totally unknown. For the situation of a multivariate process with an unknown distribution, Yue and Liu^5^, from the point of Mahalanobis data depth, introduced a chart for monitoring processes for multivariate process. Data depth is efficient and totally nonparametric. However, the computational complexity is high as the number of variables grows and may influence the performance of detection of a chart. In addition, the covariance matrix of the data depth method is constant^5^. Therefore, the method may be unsuitable when the covariance matrix in a process is not stable. Zou and Tsung^6^ proposed a new multivariate EWMA chart to detect location parameters. The chart is affine-invariant, and its controlled run length distribution is the same for the class of distributions with elliptical directions.

Some strictly distribution–free rank–based methods have been developed to increase the efficiency in detecting a nonparametric process^7–9^. The computation speed of these rank–based methods is fast, and the methods are easy to implement. However, all of these methods focus on a univariate process. In our article, we introduce a new nonparametric multivariate EWMA chart based on rank method, which is combined with the Hotelling *T*^2^ statistic for a multivariate process. This method is completely distribution–free, and it is easy to implement in applications. Moreover, the covariance matrix of observations keeps being updated as new observations arrive. Additionally, the computation load is very light.

For multivariate or high-dimensional statistical process control, location parameter shifts sometimes occur in only one or a few characteristics in a process. We want to detect these shifts quickly, accurately and to identify the shifted location parameter components. Consider this issue, fruitful nonparametric control charts have been introduced in the literature. Qiu and Hawkins^10^ and Hawkins^11^ constructed a new multivariate statistical process control chart and indicated that proposed chart was more efficient than the *T*^2^ control chart when a shift occurred in only one characteristic. However, the shift of a process is usually unknown and may occur in several highly correlated variables. To address this issue, in the context of a process where the location parameter often changes in a few number of variables, Zou and Qiu^12^ proposed a useful multivariate statistical process control chart by using the LASSO tool. In addition, inspired by Zou and Tsung, Liang *et al*.^13^ came up with a new multivariate EWMA chart to monitor sparse mean changes. In our paper, the proposed method is designed to detect sparse mean changes, and the results shows that this method performs relatively better in applications.

Previous studies showed that the multivariate control chart could be useful for biosurveillance. Rogerson and Yamada^14^ proposed a multivariate cumulative sum approach to detect the change in spatial patterns and applied it to a county-level breast cancer datasets. Their results suggested that the proposed chart for multivariate process performed relatively better compared with the univariate method when shifts occurred in many regions. Abdollahian and Hayati Rezvan^15^ applied a multivariate EWMA control chart to monitor patient’s progress after cardiac surgery, in which the proposed multivariate EWMA chart can detect an out-of-control signal that was missed by the univariate EWMA charts. This is because that the correlations between charting statistics are ignored in univariate chart. Then the univariate chart may give a misleading indication when such correlation is considerably high.

The structure of this paper is organized as follows: in Section 2, the rank–based method is given, and a nonparametric chart for online monitoring is provided. A simulation of this control chart is presented in Section 3. Real data are studied to illustrate the performance of the proposed control chart in Section 4. Finally, some conclusions are presented in Section 5.

## Model

### EWMA control chart

The EWMA control chart has good properties for control applications. Lucas and Saccucci^16^ studied the performance of EWMA and CUSUM charts. In their paper, the EWMA chart has relatively better performance for small shifts with an appropriate smoothing parameter. The EWMA control chart is first introduced for univariate variables. The EWMA control chart is easy to construct and implement, and it is based on the following statistic:

$${Z}_{i}=\lambda {X}_{i}+(1-\lambda ){Z}_{i-1},\,0 < \lambda \le 1,$$

*Z*_*i*_ is the EWMA statistic, where the starting value is *Z*_0_ = 0, and *λ* is a smoothing parameter. *X*_*i*_ represents the observations in a process. The EWMA chart corresponds to a Shewhart control chart when *λ* = 1. The weight of the historical data is decided by the magnitude of the smoothing parameter. A process is considered out-of-control (OC) whenever *Z*_*i*_ falls outside the range of the control limits.

### Rank–based methods

A rank–based method is first given for a one–dimensional process. Liu *et al*.^9^ introduced the rank–based method and assumed that independent observations, *X*_*i*_, follow the model below:

$${X}_{i} \sim \{\begin{array}{cc}F(X,{\mu }_{0}), & if\,i=1,2,\cdots ,\tau ,\\ F(X,{\mu }_{1}), & if\,i=\tau +1,\tau +2,\cdots ,\end{array}$$

where *μ*_0_ is the in-control (IC) location parameter, and *μ*_1_ is the OC location parameter. *τ* is the unknown change point. *F* is an unknown continuous distribution function. Let *R*_*i*_ denote the *i* th sequential rank; Liu *et al*.^9^ presented the formula for the rank of *X*_*i*_ among *X*_1_, *X*_2_, …, *X*_*i*_, …, *X*_*n*_ as follws:

$${R}_{i}=\mathop{\sum }\limits_{j=1}^{i}\,I\{{X}_{i}\ge {X}_{j}\}.$$

The standardized sequential rank was defined as

$${R}_{i}^{\ast }=\frac{{R}_{i}-E{R}_{i}}{\sqrt{Var{R}_{i}}}(i\ge 2),$$

where

$$E{R}_{i}=\mathop{\sum }\limits_{r=1}^{i}\,r\times P({R}_{i}=r)=\mathop{\sum }\limits_{r=1}^{i}\,r\times \frac{1}{i}=\frac{i(i+1)}{2}\times \frac{1}{i}=\frac{i+1}{2},$$

$$Var{R}_{i}=E({R}_{i}^{2})-{(E({R}_{i}))}^{2}=\mathop{\sum }\limits_{r\mathrm{=1}}^{i}\,{r}^{2}\times P({R}_{i}=r)-{(\frac{i+1}{2})}^{2}=\frac{(i+\mathrm{1)(}i-\mathrm{1)}}{12}\mathrm{.}$$

$${R}_{i} \sim U[1,\,i]$$. Therefore,

$$({R}_{i}-\frac{i+1}{2})/\sqrt{\frac{(i+\mathrm{1)(}i-\mathrm{1)}}{12}} \sim U[(1-\frac{i+1}{2})/\sqrt{\frac{(i+\mathrm{1)(}i-\mathrm{1)}}{12}},(i-\frac{i+1}{2})/\sqrt{\frac{(i+\mathrm{1)(}i-\mathrm{1)}}{12}}]\mathrm{.}$$

Then,

$$(1-\frac{i+1}{2})/\sqrt{\frac{(i+\mathrm{1)(}i-\mathrm{1)}}{12}}=(\frac{1-i}{2})/\sqrt{\frac{(i+\mathrm{1)(}i-\mathrm{1)}}{12}}=-\sqrt{\mathrm{3((}i-\mathrm{1)/(}i+\mathrm{1)}},$$

$$(i-\frac{i+1}{2})/\sqrt{\frac{(i+\mathrm{1)(}i-\mathrm{1)}}{12}}=(\frac{i-1}{2})/\sqrt{\frac{(i+\mathrm{1)(}i-\mathrm{1)}}{12}}=\sqrt{\mathrm{3((}i-\mathrm{1)/(}i+\mathrm{1)}}\mathrm{.}$$

Therefore, the distribution of *R*_i_^*^ is defined in the interval

$$[-\sqrt{\mathrm{3((}i-\mathrm{1)/(}i+\mathrm{1)}},\sqrt{\mathrm{3((}i-\mathrm{1)/(}i+\mathrm{1)}}].$$

The asymptotic distribution of *R*_i_^*^ is U($-\sqrt{3}$, $\sqrt{3}$) as *i* → ∞.

In the context of a multivariate process, it is supposed that there are *m* independent observations from an unknown multivariate continuous distribution with dimensionality *p*. That is, *Y*_*i*_ = (*Y*_1,*i*_,*Y*_2,*i*_, …, *Y*_*p*,*i*_)′, *i* = 1, 2, …, *m*. There are *p* characteristics to be examined that we are interested in. For a set of variables, *Y*_*j*,1_, *Y*_*j*,2_, …, *Y*_*j*,*m*_, *j* = 1, 2, …, *p*, which represents the *j* th characteristic with *m* observations, the rank–based method can be used to construct statistics. When the observations are *p*-dimensional, the *i* th observations are *Y*_*i*_ = (*Y*_1,*i*_, *Y*_2,*i*_, …, *Y*_*p*,*i*_)′. For the *j* th component, *Y*_*j*,*i*_, *R*_*j*,i_^*^ denote the *i* th standardized sequential rank with the arrival of the *j* th component *Y*_*j*,*i*_. Therefore, the vectors *Q*_*i*_ = (*R*_1,i_^*^, *R*_2,i_^*^, …, *R*_*p*,i_^*^)′ can be obtained. In addition, each component *R*_*j*,i_^*^ follows the same uniform distribution as *R*_i_^*^. Then, the EWMA statistics can be constructed, which are based on *T*^2^ statistics. The EWMA statistics are given by

$${Z}_{i}=R{Q}_{i}+(I-R){Z}_{i-1},$$

where R = diag(*λ*_1_, *λ*_2_, …, *λ*_*k*_, …, *λ*_*p*_), <*λ*_*k*_ ≤ 1 represents the smoothing parameter. *I* represents the *p*-dimensional identity matrix. If there is no a priori information given, different smoothing parameters are needed for different components; then, *λ*_1_ = *λ*_2_ = ·*·*· = *λ*_*k*_ = ··· = *λ*_*p*_ are used, and the starting value is *Z*_0_ = (0, 0, …, 0)′. The process is considered to be OC if a manufacturing or business process is in a state of uncontrollable (i.e. *Z*_*i*_^Τ^Σ_*Zi*_^−1^*Z*_*i*_ > *L*), where *L* is the upper control limit. And the covariance matrix of *Z*_*i*_ is as follows:

$${\varSigma }_{{Z}_{i}}=\mathop{\sum }\limits_{k=1}^{i}\,R{(I-R)}^{i-k}\varSigma {(I-R)}^{i-k}R\mathrm{.}$$

In particular, Σ_*Zi*_ = (1−(1−*λ*)^2*i*^)*λ*/(2−*λ*)Σ when *λ*_1_ = *λ*_2_ = ··· = *λ*_*k*_ = ··· = *λ*_*p*_ = *λ*. *λ* is a fixed value. Usually, we take the limit form, Σ_*Zi*_ = *λ*/(2−*λ*)Σ. Σ, the covariance matrix of *Q*_*i*_, is estimated from samples in practice.

## Simulation

In the art of research, fruitful distribution–free control charts have been introduced. If a chart IC run–length distributions are the same to every continuous distribution^17^, we call this chart is nonparametric or distribution-free. We discuss the choice of parameter by using the multivariate normal distribution. This indicates that the determine of parameters are still valid when a series of observations obey other distributions. Therefore, we consider the *i* th observation, *X*_*i*_, is collected as time goes by using the following relational model:

$${X}_{i} \sim \{\begin{array}{cc}N({\mu }_{IC},{\varSigma }_{IC}\mathrm{),\ } & i=\mathrm{1,}\,\mathrm{2,}\cdots ,\tau ,\\ N({\mu }_{OC},{\varSigma }_{IC}), & i=\tau +\mathrm{1,}\,\tau +\mathrm{2,}\cdots ,\end{array}$$

where

$${\mu }_{IC}=(0,0,0),\,{\varSigma }_{IC}=(\begin{array}{ccc}1 & 0 & 0\\ 0 & 1 & 0\\ 0 & 0 & 1\end{array})\,and\,{\mu }_{OC}=(\delta ,0,0).$$

And *α* is the probability of a type I error and *β* is the probability of a type II error. For a fair comparison, we usually fix *α* and compare *β*. A small *β* is considered better. The average run length (ARL) is the number of points that, on average will be plotted on a control chart before an OC signal. If a manufacturing or business process is IC:

$$AR{L}_{0}=1/\alpha .$$

If the process is considered OC:

$$AR{L}_{1}=1/(1-\beta ).$$

Therefore, we fix IC ARL, *ARL*_0_ and compare OC ARL, *ARL*_1_. A small *ARL*_1_ is considered better.

Meanwhile, inspired by Han and Tsung^18^, we consider the relative mean index (RMI) values to evaluate the average performance of these charts for detecting a range of parameter changes, which are given as following:

$${\rm{RMI}}=\frac{1}{m}{\sum }_{i\mathrm{=1}}^{m}\,\frac{AR{L}_{{\delta }_{X}}-MAR{L}_{{\delta }_{X}}}{MAR{L}_{{\delta }_{X}}},$$

where m is the number of shifts that we considered. When detecting a certain shift *δ*_*X*_, *ARL*_*δX*_ denotes as the OC ARL of these given charts. And *MARL*_*δX*_ is the smallest OC ARL among all the OC ARL values of these charts when detecting a certain shift *δ*_*X*_. The RMI calculates the average of all the detection efficiency values^18^. A control chart with a relatively smaller RMI value is regarded as relatively better detection efficiency.

We suppose that there are 1000 independent and identically distributed historical (reference) observations. *X*_1_, *X*_2_, …, *X*_1000_ are 1000 random observations from *N*(*μ*_0_,Σ_0_). To make a fair comparison, all of these control charts have the same IC zero–state ARL, which is equal to 500. It should be note that zero-state run lengths refer to the run lengths of control charts initialized at the target value^16^. When the process goes OC, a chart is considered as a better detection efficiency with a small ARL. The ARLs of these EWMA methods with *λ* = 0.03 for a range of shifts are presented in Table 1. *EWMA*_1_ represents the rank-based EWMA scheme, and *EWMA*_2_ represents an EWMA control chart based on the Mahalanobis depth method^5^. We also provide simulation studies with the non-diagonal covariance matrix

$${\varSigma }_{1}=(\begin{array}{ccc}9 & 8 & 8\\ 8 & 9 & 8\\ 8 & 8 & 9\end{array}),$$

**Table 1:** ARL comparisons for the EWMA control chart under *N*(*μ*_0_,Σ_0_) with a zero–state ARL = 500.

	*δ*_*X*_								RMI
	0.25	0.5	1	1.5	2	2.5	3	4	
***τ*** **= 100**									
*EWMA*_1_	210.9	91.4	25.8	15.9	11.6	10.4	10	9.6	0.04
*EWMA*_2_	325.7	154.9	39.8	19.6	12.9	10.1	8.9	8	0.27
***τ*** **= 200**									
*EWMA*_1_	108.7	63.2	33.3	20	12.3	11	10.7	9.5	0.16
*EWMA*_2_	314	147.7	34.9	17.2	11.2	8.7	7.6	6.1	0.41
***τ*** **= 400**									
*EWMA*_1_	137.3	76.1	37.4	20.2	15.2	12.7	11.8	10.3	0.24
*EWMA*_2_	347.7	145.4	38.1	18.1	11.3	9	7.7	6.9	0.31

The ARLs of the EWMA scheme with *λ* = 0.03 for a range of shifts under *N*(*μ*_0_,Σ_1_) are presented in Table 2. In addition, the detection performance of these charts under a bivariate Weibull distribution, *LBVW*(*θ*_1_, *θ*_2_,*α*,*ρ*) are shown in Table 3. *θ*_1_ and *θ*_2_ are the scale parameter. *α* is the shape parameters. *ρ* is the correlation coefficient. When a process is IC, $({X}_{1},{X}_{2}) \sim LBVW({\theta }_{1},{\theta }_{2},\alpha ,\rho )$. $({X}_{1},{X}_{2}) \sim LBVW({\theta }_{1},{\theta }_{2},\alpha +{\delta }_{X},\rho )$ when the process is OC. Tables 1–3 provide the ARL of the *EWMA*_1_ and *EWMA*_2_ control charts for a range of shifts *δ*_*X*_. Tables 1–3 show that the *EWMA*_1_ control chart has a relatively better performance for detecting small shifts. *EWMA*_2_ has a better performance for detecting large shifts. On the whole, *EWMA*_1_ has a relatively small RMI.

**Table 2:** ARL comparisons for the EWMA control chart under *N*(*μ*_0_,Σ_1_) with a zero–state ARL = 500.

	*δ*_*X*_								RMI
	0.25	0.5	1	1.5	2	2.5	3	4	
***τ*** **= 100**									
*EWMA*_1_	239.1	177.5	28.1	16.5	12.9	11.5	10.3	9.1	0.02
*EWMA*_2_	345.2	281.5	47	24.3	15.7	11.3	9.6	8.4	0.3
***τ*** **= 200**									
*EWMA*_1_	211.6	127.4	27.6	16	12.8	10.5	9.6	7.9	0.04
*EWMA*_2_	260.5	163.5	45.8	23.7	15.1	10	8.3	7	0.23
***τ*** **= 400**									
*EWMA*_1_	190	94	26.3	15.5	12.9	9.8	8.9	7.5	0.04
*EWMA*_2_	236.9	149.6	41.3	21.6	14.7	9.1	7.5	6.9	0.24

**Table 3:** ARL comparisons for the EWMA control chart under *LBVW*(1, 1, 1, 0.5) with a zero–state ARL = 500.

	*δ*_*X*_								RMI
	0.25	0.5	1	1.5	2	2.5	3	4	
***τ*** **= 100**									
*EWMA*_1_	132.7	62.1	24.2	16.7	13.9	12.9	11.8	10.6	0.1
*EWMA*_2_	156	94.9	40	19	12.3	10.1	9.9	9	0.19
***τ*** **= 200**									
*EWMA*_1_	116.2	42.9	23.7	16.2	13.9	12.8	11.8	10.3	0.11
*EWMA*_2_	134	62	31.9	21	11.2	10	9.6	8.9	0.16
***τ*** **= 400**									
*EWMA*_1_	93.3	39.3	21.7	16.2	13.1	11.9	11.5	10.1	0.1
*EWMA*_2_	107.7	55.4	26	18.1	11	10.2	9.7	8.1	0.11

Table 4 presents the simulation results under *N*(*μ*_2_,Σ_2_), where *μ*_2_ = (0, 0, 0, 0, 0, 0) and Σ_2_ is 6 × 6 indentity matrix. Table 4 shows that *EWMA*_1_ still performs better. Sometimes, we encounter the case that observations follow block-diagonal correlation structures. Therefore, we provided ARL comparisons for observations follow a block-diagonal correlation structures, which presented in Table 5. Where *μ*_3_ = (0, 0, 0, 0) and

$${\varSigma }_{3}=(\begin{array}{cccc}1 & 1 & 0 & 0\\ 1 & 3 & 0 & 0\\ 0 & 0 & 1 & 1\\ 0 & 0 & 1 & 2\end{array})\mathrm{.}$$

**Table 4:** ARL comparisons for the EWMA control chart under *N*(*μ*_2_,Σ_2_) with a zero–state ARL = 500.

	*δ*_*X*_								RMI
	0.25	0.5	1	1.5	2	2.5	3	4	
***τ*** **= 100**									
*EWMA*_1_	135.4	37.4	15.3	12.8	9.4	8.9	8	7.6	0.04
*EWMA*_2_	175.2	61.5	24	13.3	8.7	8.2	7.6	7	0.19
***τ*** = 200									
*EWMA*_1_	85	28.4	15	11.2	9	8.1	7.5	7.1	0.03
*EWMA*_2_	106.5	43.5	19.9	12.7	8.1	7.5	7.3	7	0.16
***τ*** **= 400**									
*EWMA*_1_	70.9	21.7	13.5	10.9	8.6	7.5	7.3	7	0.03
*EWMA*_2_	89.9	38.6	16.3	11.6	8	7.1	7	6.8	0.12

**Table 5:** ARL comparisons for the EWMA control chart designed to detect a shift under *N*(*μ*_3_,Σ_3_) with a zero–state ARL = 500.

	*δ*_*X*_								RMI
	0.25	0.5	1	1.5	2	2.5	3	4	
***τ*** **= 100**									
*EWMA*_1_	114.9	31.1	13.7	11.3	9.9	8.9	8.6	7.9	0.01
*EWMA*_2_	341	122	31.6	14.5	11.7	8.8	8.4	7.5	0.83
***τ*** **= 200**									
*EWMA*_1_	78.9	29.3	13.4	10.4	9.3	8.5	7.1	7.1	0.02
*EWMA*_2_	198.7	98.3	28.7	13.8	10.5	8.5	7.1	6.1	0.68
***τ*** **= 400**									
*EWMA*_1_	67.6	26.3	12.9	9.3	8.7	8.1	7.1	7.1	0.02
*EWMA*_2_	110.6	68	21.9	11.6	8.5	7.9	6.8	6.8	0.4

Table 5 shows the proposed methods performs relatively better. In addition, the proposed control chart based on ranks of data is a nonparametric method without assuming normal or Poisson distribution for the data. To investigate the performance of the proposed method for Poisson data, we conducted an additional simulation study under multivariate Poisson distribution. Results in Table 6 showed that the proposed methods (*EWMA*_1_) still had a better performance in terms of the OC ARL and RMI.

**Table 6:** ARL comparisons for the EWMA control chart designed to detect a shift under multivariate Poisson(*θ*_1_ + *δ*_*X*_, *θ*_2_, *θ*_0_) with a zero–state ARL = 500, where (*θ*_1_, *θ*_2_, *θ*_0_) = (0.5, 0.6, 0.2).

	*δ*_*X*_								RMI
	0.25	0.5	1	1.5	2	2.5	3	4	
***τ*** **= 100**									
*EWMA*_1_	105.2	29.7	13.6	12.2	10.9	9.3	8.9	8.1	0.02
*EWMA*_2_	111.7	31.2	16	12.7	10.6	8.5	8.5	7.9	0.04
***τ*** **= 200**									
*EWMA*_1_	66.4	25	13.3	10.3	9	8.6	8.1	7.9	0.03
*EWMA*_2_	71.7	27.9	14.4	10.6	9.1	8.1	7.3	7.2	0.04
***τ*** **= 400**									
*EWMA*_1_	52.6	24.9	13.1	10.7	8.6	8.3	8.1	7.7	0.02
*EWMA*_2_	61.8	25.5	14.1	10.9	8.3	8.3	7.6	7.3	0.04

**Figure 1:**
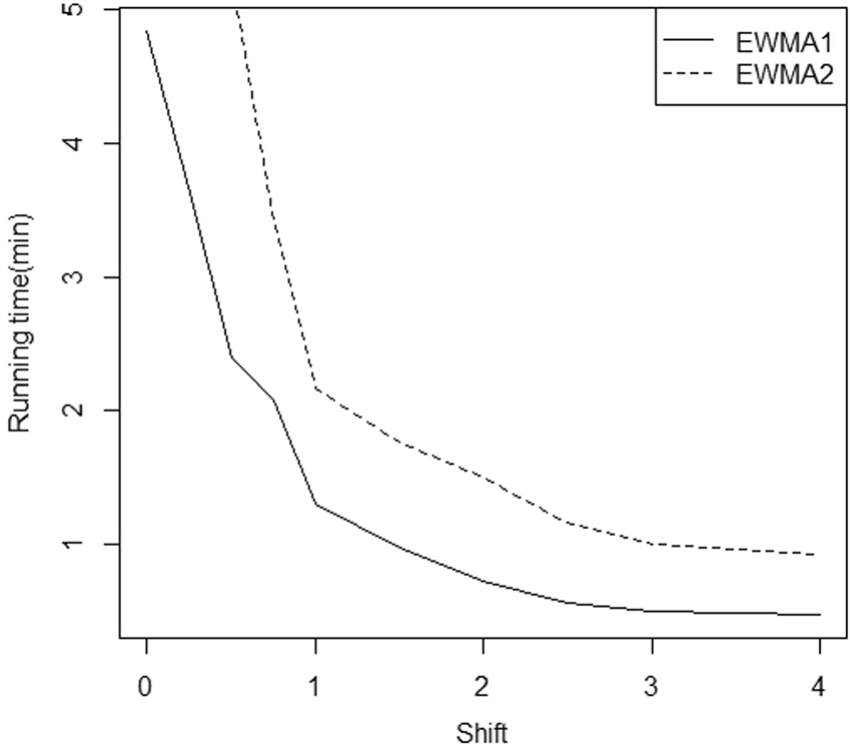
Computing time of the *EWMA*_1_ and *EWMA*_2_ charts for a range of shifts.

In addition, we also provide the computing time of the *EWMA*_1_ and *EWMA*_2_ control charts. From Fig. 1, *EWMA*_1_ has relatively shorter computing time compared to that of *EWMA*_2_. Therefore, the proposed EWMA control chart is chosen, which is based on rank methods, for monitoring in this paper.

## Analysis of Japanese Data

### Data source

That is the case, with the Japanese influenza data^19^, which cover 6 regions in Japan. These regions include *Gunma*, *Chiba*, *Tokyo*, *Ishikawa*, *Nagano*, and *Osaka*. Influenza data analysis is a very important issue today^20,21^. Simultaneous monitoring of flu break–outs in multiple regions is an important topic in epidemiology. Influenza is an acute contagious disease caused by a virus^19^. The Japanese influenza data are used to illustrate the proposed control chart. Time–series data of the weekly incidence of influenza in Japan are used from January 2000 through December 2011. To evaluate the incidence data (see “Influenza Dataset” in Supplementary Information), we conduct spectral analysis, which is useful for investigating the periodicities of shorter time series, such as that of the incidence data used in the present study.

**Figure 2:**
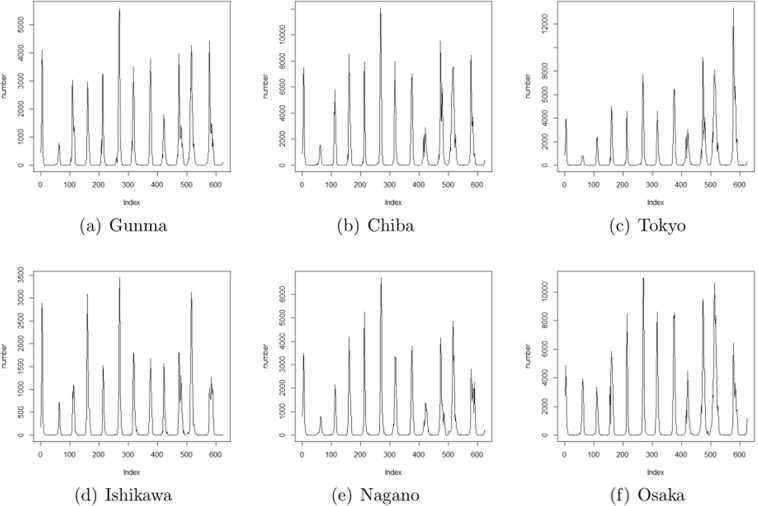
The Japanese influenza data.

**Figure 3:**
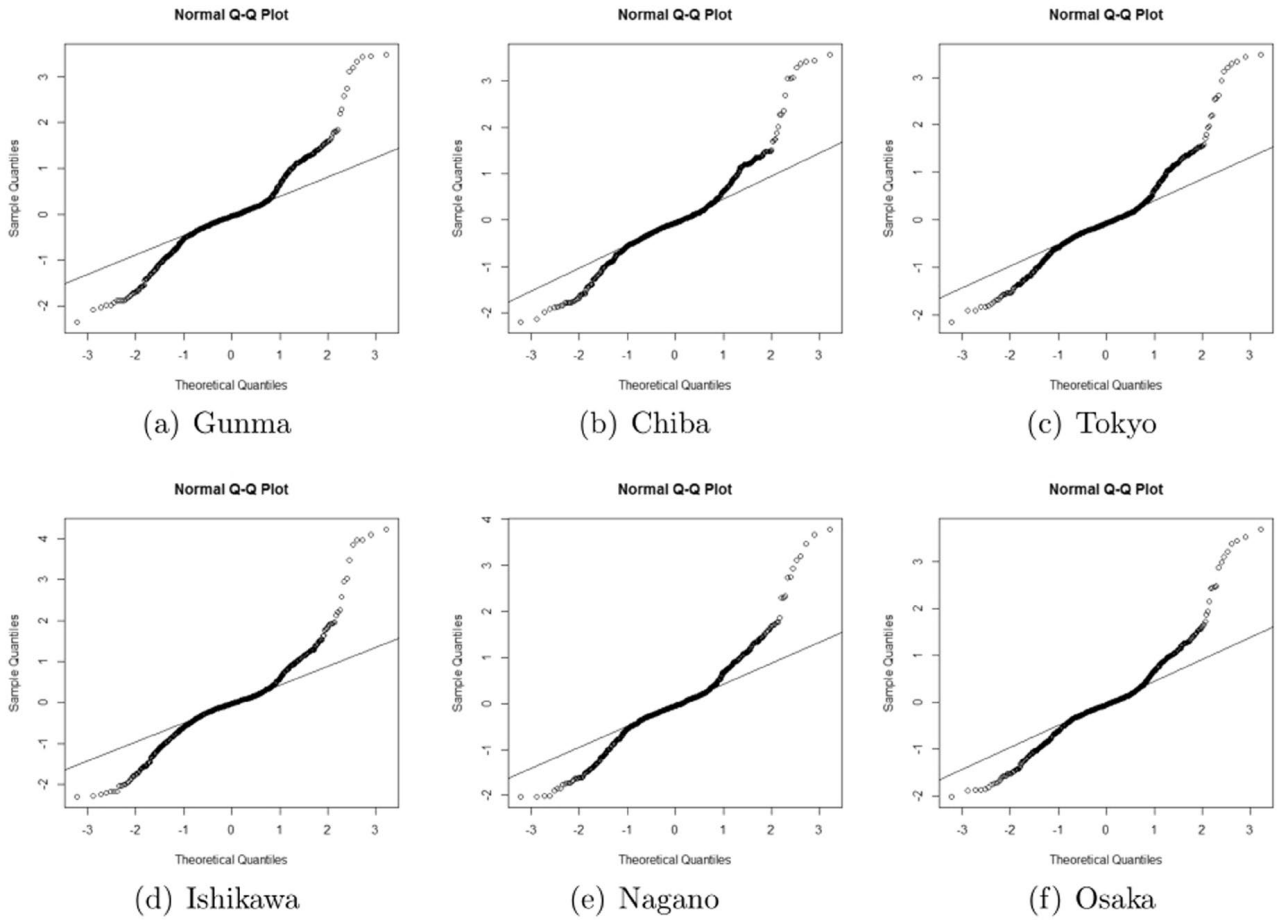
The corresponding normal Q-Q plot.

The Japanese influenza data are presented in Fig. 2. A quantile–quantile (Q–Q) plot of each region that includes 782 historical observations is presented in Fig. 3. Figure 3 suggests that the normality assumption for the influenza data is invalid.

**Figure 4:**
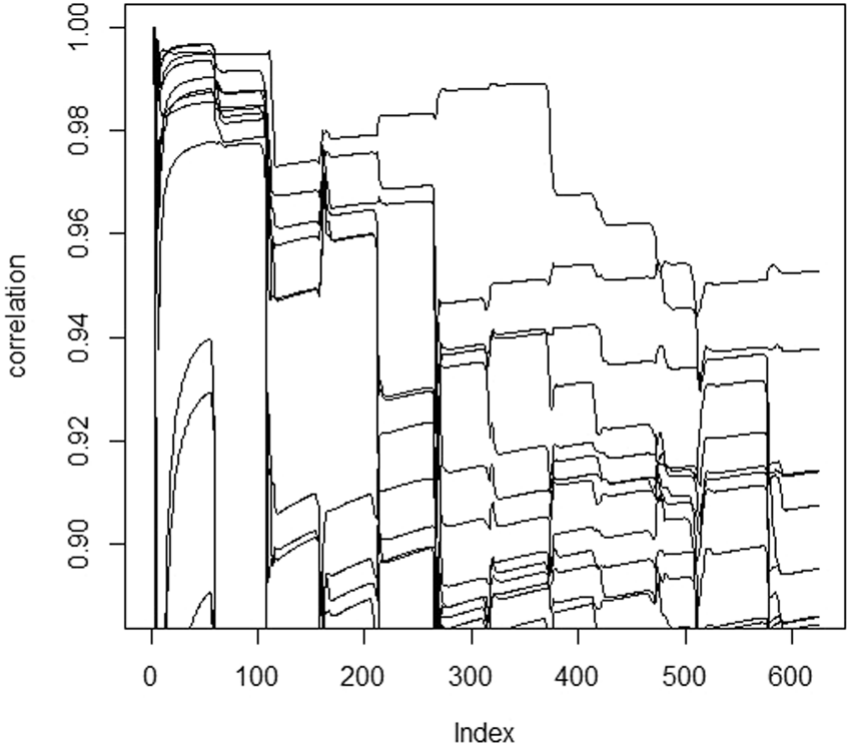
Correlation of six regions.

The correlation of six regions as shown in Fig. 4, for a total of *C*_6_^2^ = 15 lines. Figure 4 shows that the cross-correlation is not stable. Therefore, we update the covariance matrix with the arrival of new observations. It should be noted that the covariance matrix Σ is updated, as presented in section 2.2.

### Data analysis

In this section, a multivariate control chart is used to monitor the incidence of influenza in six regions which may have a certain correlation. Ignoring the correlation and using several univariate charts could lead to biased conclusions. For example, the univariate chart statistic may result in unnecessarily frequent out-of-control signals when the process is actually in control and may not detect the change when the process becomes out of control^3^.

In the past few decades, many researchers have studied spectral analysis^22^. In addition to the obvious annual cycle of influenza epidemics, the longer–term incidence patterns are important for interpreting the mechanism of influenza epidemics. The method proposed by Sawada *et al*.^23^ is a combination of spectral analysis and non–linear least squares fitting (LSF) for fitting analysis. Spectral analysis is a useful tool to investigate the periodicities of a short time series, and the formulations of the LSF curve are related to the research of Sawada *et al*.

Spectral analysis is used identify the interepidemic period of influenza epidemics in Japan (see “Computing Code” in Supplementary Information). Based on spectral analysis, the trend of the incidence data is determined. The procedure comprises the following 3 steps. In step I, the influenza data (standardized datasets) are preprocessed. In step II, the temporal behavior of the interepidemic period is investigated. Then, LSF is used for the fitting analysis. This trend is then removed by subtracting the LSF curve from the data, thereby yielding the residual time–series data. In step III, the obtained residual time–series datasets are analysed.

**Figure 5:**
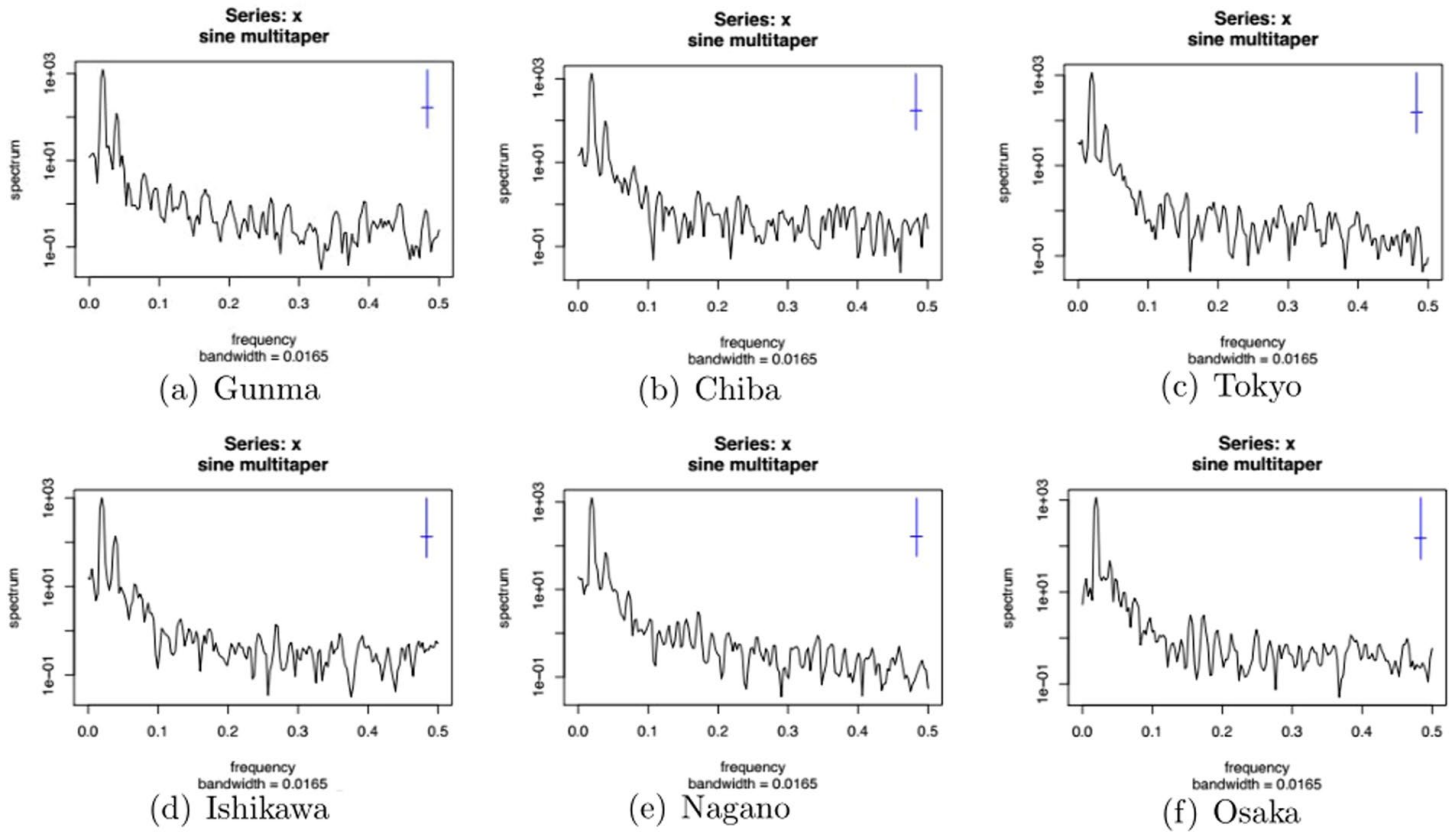
Spectral analysis of the influenza data series.

The vertical coordinates of Fig. 5 represents the power spectral density (PSD). Figure 5 indicates that the numbers of the maximum entropy method (MEM) spectral periods. From Fig. 5 and the processed data, we find that the power has a large magnitude at a frequency of 0.035 (1/week), and there is a second peak at a frequency of 0.019 (1/week). A large magnitude indicates that a large portion of the amplitude of the incidence data is expressed as a wave that repeats itself every year. Spectral analysis has enabled us to identify multiple periodicities for the interepidemic period of influenza epidemics (1- and 0.5-year periods). The residual time–series data are relevant.

For residuals data, Table 7 presents the results of Shapiro-Wilk test and Kolmogorov-Smirnov test for normality. The *p*-values are smaller than 0.05, indicating that the data are non-normally distributed. Therefore, a nonparametric control chart could be more appropriate than those based on normality assumption. Moreover, a first order autoregressive model (AR(1)) is used to analyze the sequence correlation. Table 8 shows that sequences are highly correlated. Thus, the first order difference is employed to reduce the sequence correlation (see results in Table 9). Then the differential data can be used to illustrate the proposed method.

**Table 7:** Shapiro-Wilk test and Kolmogorov-Smirnov test for normality.

	Gunma	Chiba	Tokyo	Ishikawa	Nagano	Osaka
**Shapiro-Wilk test**						
*W*	0.95738	0.962	0.98165	0.93915	0.95504	0.94605
*p*−*value*	2.752e-14	2.271e-13	2.464e-08	<2.2e-16	1.002e-14	2.809e-16
**Kolmogorov-Smirnov test**						
*D*	0.075224	0.12162	0.17872	0.10759	0.071647	0.10472
*p*−*value*	0.0002868	1.796e-10	<2.2e-16	2.747e-08	0.000652	7.112e-08

**Table 8:** The coefficients of AR(1) for residuals data.

	Gunma	Chiba	Tokyo	Ishikawa	Nagano	Osaka
Coefficients	0.9086	0.9105	0.9364	0.8854	0.9039	0.9111

**Table 9:** The coefficients of AR(1) for residuals data after the first order difference.

	Gunma	Chiba	Tokyo	Ishikawa	Nagano	Osaka
Coefficients	−0.1249	−0.1813	−0.1563	−0.2178	−0.0699	−0.2118

**Figure 6:**
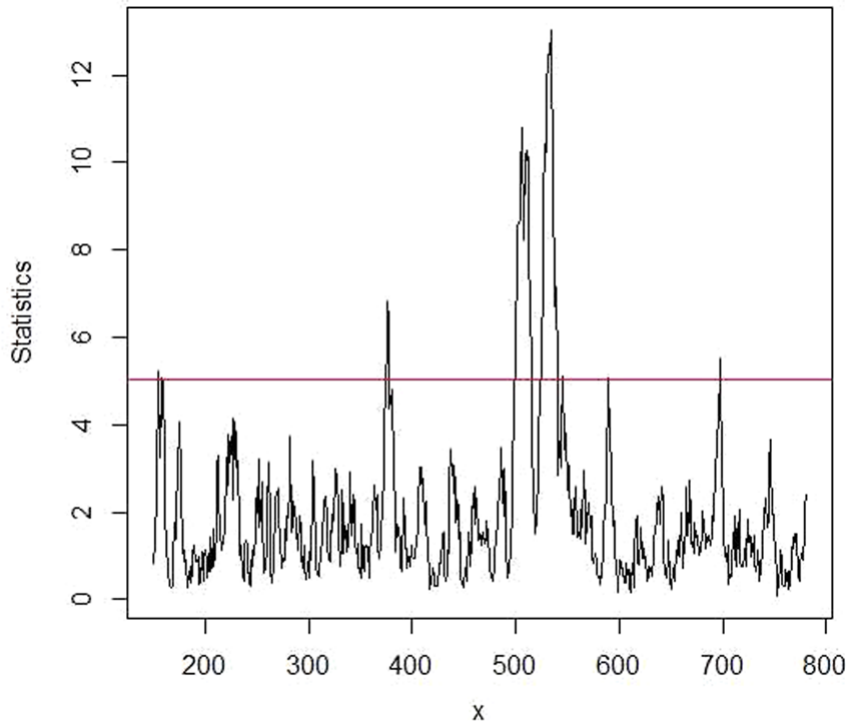
*EWMA*_1_ control chart.

**Figure 7:**
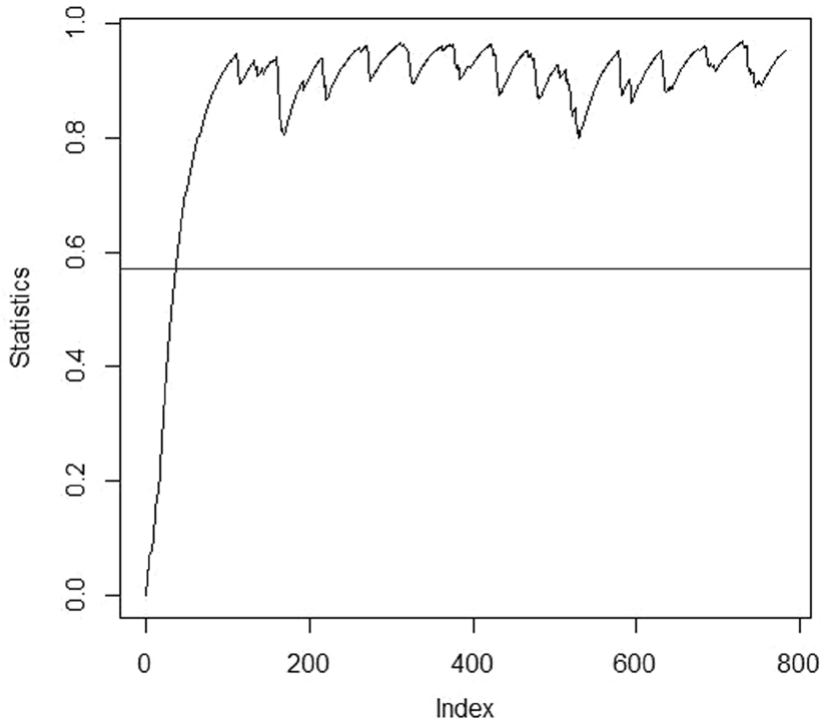
EWMA control chart based on data depth.

The *EWMA*_1_ control chart of the residual data series is presented in Fig. 6. Figure 6 shows that EWMA statistics fall outside the range of the control limits in 2003, 2006, 2009. SARS jumped simultaneously from a village in China to two cities on opposite sides of the world, Singapore and Toronto, in 2003. H5N1 outbreaks in poultry peaked in 2006, and the highly pathogenic H5N1 avian influenza virus spread to affect wild or domestic birds in 17 new countries in Africa, Asia, Europe, and the Middle East. The H1N1 influenza pandemic continued to spread in 2009. From Fig. 7, the four peaks occurred at approximately the 160th case (2003-1-19), 366th case (2006-12-31), 509th case (2009-9-27), and 596th case (2011-5-29), respectively. The signal of alarm appeared for the 159th case (2003-1-12), 363th case (2006-12-10), 502th case (2009-8-9), suggesting that the proposed method can provide early detection of influenza epidemics.

We provide the performance of *EWMA*_2_ by using Japanese influenza data (Fig. 7). It can be observed that the *EWMA*_2_ chart shows an inconsistent trend with the result in practice (the charting statistics indicate that the six regions are almost at the epidemic level after 32 cases). This may be caused by the constant covariance setting in *EWMA*_2_. Hence, updating the covariance between the six regions could be important in correctly detecting an epidemic of influenza.

**Figure 8:**
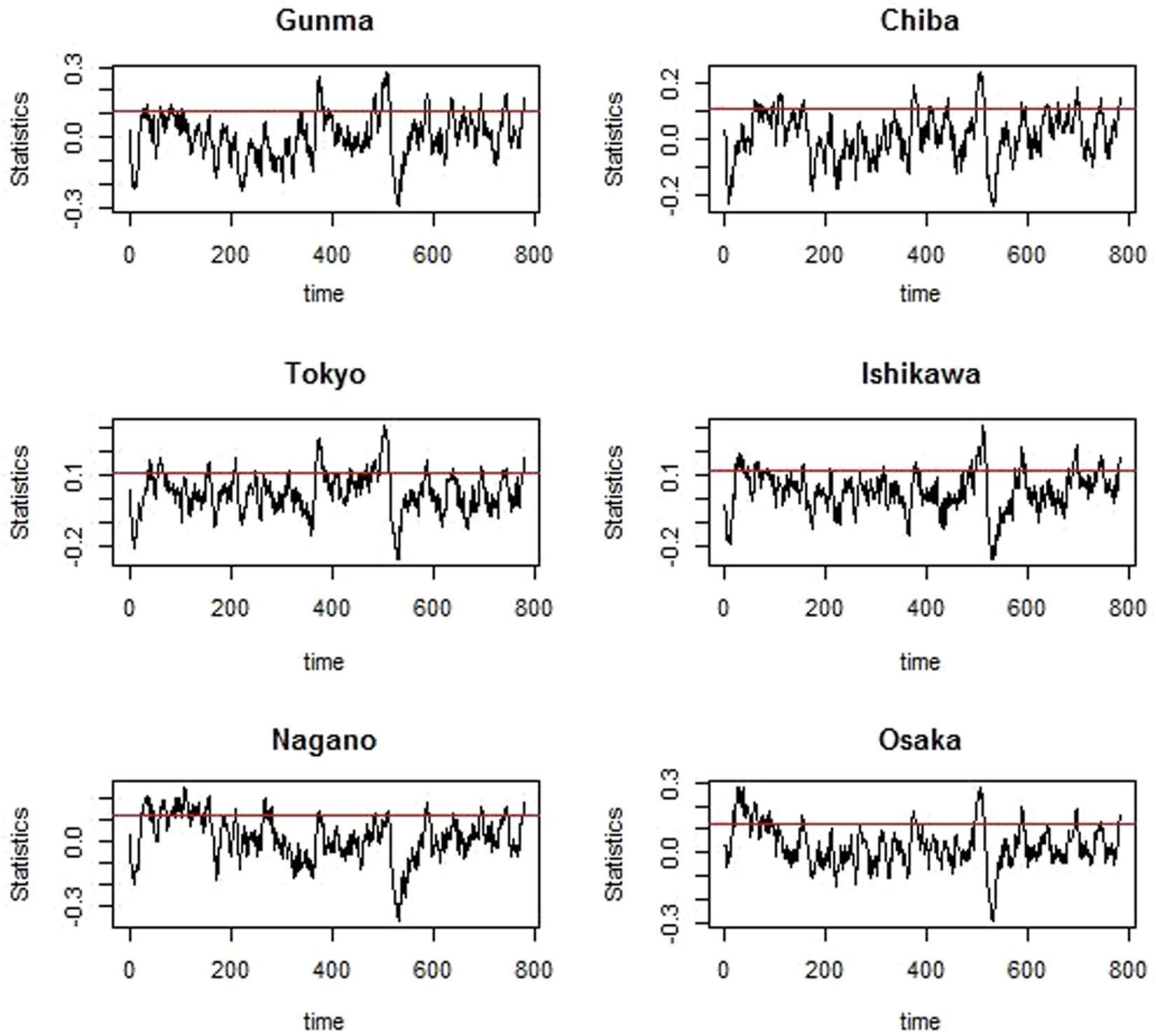
Six single univariate control charts for Japanese influenza data.

We also presented six single univariate control charts for Japanese influenza data in Fig. 8. The univariate chart statistic gave unnecessarily frequent out-of-control signals when the process is actually in control. Specifically, the first out-of-control signal of six regions occurred approximately at the 30th case, the 61th case, the 42th case, the 24th case, the 27th case, and the 17th case, respectively. However the multivariate chart may suggest a in-control state, indicating that ignoring the correlation between regions in biosurveillance may give an unexpected high rate of false alarm.

## Conclusions

This paper provides a new EWMA control chart based on rank methods for a multivariate process. The performance of an EWMA control chart based on rank methods and Mahalanobis depth are compared. The EWMA control chart based on rank methods has a relatively better performance for detecting small shifts. Finally, Japanese influenza data are also provided to illustrate the proposed control chart. Spectral analysis is first conducted to investigate the periodicities of shorter time series, and then non–linear least squares fitting is used for fitting analysis. The residual data series are obtained, and the residual data series are monitored. The Japanese influenza data example shows that the proposed control chart has relatively better performance for detecting process changes.

## Supplementary information

Computing Code

Influenza Dataset

## Data Availability

The datasets analyzed during the current study are available from the corresponding author on reasonable request.
