# Supplementary material for: Multivariate nonparametric chart for influenza epidemic monitoring
Source: Sci Rep. 2019 Nov 25;9:17472. doi: 10.1038/s41598-019-53908-6 (PMC6877522; doi:10.1038/s41598-019-53908-6)
Supplement: Supplementary file 1 — Computing Code [file 41598_2019_53908_MOESM1_ESM.pdf]

Multivariate nonparametric chart for influenza epidemic monitoring

Liu Liu, Jin Yue, Xin Lai, Jian Zhang

The following R code is used to find the control limits and ARL in Table 5, and to perform the spectral analysis in Figure 6, the correlation analysis in Figure 4 and the Q-Q plot in Figure 3

code

```
#Step I
#Power spectral density (Figure 6), Partial autocorrelation function (Figure 4), QQ plot(Figure 3)
rm(list=ls())
library(psd)
n<-782
x1<-runif(n,0.5,1)
A<-read.table(file="C:/Users/Administrator/Desktop/Inf_Jpn_number.txt",header=T)
x<-A[,1]
x<-as.numeric(x)
Gunma<-x
par(mfrow=c(3,2))
psd=psdcore(Gunma,ntaper=2)
plot(psd)
x0<-runif(n,0.5,1)
x1<-log(x+x0)
t=c(1:length(x1))
f1=1/52;f2=1/26        #frequency
lam=data.frame(x1,a1=sin(2*pi*f1*t),b1=cos(2*pi*f1*t),a2=sin(2*pi*f2*t),b2=cos(2*pi*f2*t))
#calculate model coefficient a1, b1, a2, b2
model=lm(x1~a1+b1+a2+b2,data=lam)    #model
lambda=coef(model)%*%t(data.frame(rep(1,length(x1)),sin(2*pi*f1*t),cos(2*pi*f1*t),sin(2*pi*f2*t), cos(2*pi*f2*t)))    #established the theoretical statistical model based on lam
lambda=lambda[1,]    #convert matrix to vector
Gunma=x1-lambda    #calculate residuals
x0<-runif(n,0.5,1)
x<-A[,2]
x<-as.numeric(x)
Chiba<-x
psd=psdcore(Chiba,ntaper=2)
plot(psd)
x0<-runif(n,0.5,1)
x2<-log(x+x0)
t=c(1:length(x2))
f1=1/52;f2=1/26        #frequency
lam=data.frame(x2,a1=sin(2*pi*f1*t),b1=cos(2*pi*f1*t),a2=sin(2*pi*f2*t),b2=cos(2*pi*f2*t))#Model coefficient a1, b1, a2, b2
model=lm(x2~a1+b1+a2+b2,data=lam)    #model
lambda=coef(model)%*%t(data.frame(rep(1,length(x2)),sin(2*pi*f1*t),cos(2*pi*f1*t),sin(2*pi*f2*t), cos(2*pi*f2*t))) #established the theoretical statistical model based on lam
lambda=lambda[1,]    #convert matrix to vector
Chiba=x2-lambda #calculate residuals
x0<-runif(n,0.5,1)
x<-A[,3]
```

```
x<-as.numeric(x)
Tokyo<-x
psd=psdcore(Tokyo,ntaper=2)
plot(psd)
x0<-runif(n,0.5,1)
x3<-log(x+x0)
t=c(1:length(x3))
f1=1/52;f2=1/26      #frequency
lam=data.frame(x3,a1=sin(2*pi*f1*t),b1=cos(2*pi*f1*t),a2=sin(2*pi*f2*t),b2=cos(2*pi*f2*t))#M
odel coefficient a1, b1, a2, b2
model=lm(x3~a1+b1+a2+b2,data=lam)    #model
lambda=coef(model)%*%t(data.frame(rep(1,length(x3)),sin(2*pi*f1*t),cos(2*pi*f1*t),sin(2*pi*f2
*t), cos(2*pi*f2*t))) #established the theoretical statistical model based on lam
lambda=lambda[1,]      #convert matrix to vector
Tokyo=x3-lambda #calculate residuals
x0<-runif(n,0.5,1)
x<-A[,4]
x<-as.numeric(x)
Ishikawa<-x
psd=psdcore(Ishikawa,ntaper=2)
plot(psd)
x0<-runif(n,0.5,1)
x4<-log(x+x0)
t=c(1:length(x4))
f1=1/52;f2=1/26      #frequency
lam=data.frame(x4,a1=sin(2*pi*f1*t),b1=cos(2*pi*f1*t),a2=sin(2*pi*f2*t),b2=cos(2*pi*f2*t))#M
odel coefficient a1, b1, a2, b2
model=lm(x4~a1+b1+a2+b2,data=lam)    #model
lambda=coef(model)%*%t(data.frame(rep(1,length(x4)),sin(2*pi*f1*t),cos(2*pi*f1*t),sin(2*pi*f2
*t), cos(2*pi*f2*t))) #established the theoretical statistical model based on lam
lambda=lambda[1,]    #convert matrix to vector
Ishikawa=x4-lambda #calculate residuals
x0<-runif(n,0.5,1)
x<-A[,5]
x<-as.numeric(x)
Nagano<-x
psd=psdcore(Nagano,ntaper=2)
plot(psd)
x0<-runif(n,0.5,1)
x5<-log(x+x0)
t=c(1:length(x5))
f1=1/52;f2=1/26      #frequency
lam=data.frame(x5,a1=sin(2*pi*f1*t),b1=cos(2*pi*f1*t),a2=sin(2*pi*f2*t),b2=cos(2*pi*f2*t))#M
odel coefficient a1, b1, a2, b2
```

```
model=lm(x5~a1+b1+a2+b2,data=lam)    #model
lambda=coef(model)%*%t(data.frame(rep(1,length(x5)),sin(2*pi*f1*t),cos(2*pi*f1*t),sin(2*pi*f2
*t), cos(2*pi*f2*t))) #established the theoretical statistical model based on lam
lambda=lambda[1,]    #convert matrix to vector
Nagano=x5-lambda #calculate residuals
x0<-runif(n,0.5,1)
x<-A[,6]
x<-as.numeric(x)
Osaka<-x
psd=psdcore(Osaka,ntaper=2)
plot(psd)
x0<-runif(n,0.5,1)
x6<-log(x+x0)
t=c(1:length(x6))
f1=1/52;f2=1/26        #frequency
lam=data.frame(x6,a1=sin(2*pi*f1*t),b1=cos(2*pi*f1*t),a2=sin(2*pi*f2*t),b2=cos(2*pi*f2*t))#M
odel coefficient a1, b1, a2, b2
model=lm(x6~a1+b1+a2+b2,data=lam)    #model
lambda=coef(model)%*%t(data.frame(rep(1,length(x6)),sin(2*pi*f1*t),cos(2*pi*f1*t),sin(2*pi*f2
*t), cos(2*pi*f2*t))) #established the theoretical statistical model based on lam
lambda=lambda[1,]    #convert matrix to vector
Osaka=x6-lambda #calculate residuals
#Step II
y1<-diff(Gunma)
y2<-diff(Chiba)
y3<-diff(Tokyo)
y4<-diff(Ishikawa)
y5<-diff(Nagano)
y6<-diff(Osaka)
ar(y1, order.max = 1)
ar(y2, order.max = 1)
ar(y3, order.max = 1)
ar(y4, order.max = 1)
ar(y5, order.max = 1)
ar(y6, order.max = 1)
y<-cbind(y1,y2,y3,y4,y5,y6)
#Step III
# Rank-based multivariate control chart (Find the control limits, IC ARL=500, Table 5)
rm(list=ls())
library("MASS")    #load the package
m<-numeric(4)
m1<-numeric(4)
m2<-numeric(4)
m3<-numeric(4)
```

```
x<-numeric(4)
R1<-numeric(4)
p<-numeric(4)
Q<-numeric(4)
x2<-numeric(4)
x4<-numeric(1000)
D<-matrix
x1<-matrix(0,ncol=1000000,nrow=4)
x3<-matrix(0,ncol=4,nrow=1000000)
x8<-matrix(0,ncol=4,nrow=1000000)
z<-matrix(0,ncol=4,nrow=100000)
lambda<-0.03    #weighting parameter
I<-lambda*diag(4)
I1<-diag(4)
m<-rep(0,4)
m1<-c(0.25,0,0,0)    #in-control mean vector
D<-matrix(c(1,1,0,0,1,3,0,0,0,0,1,1,0,0,1,2),ncol=4,byrow=T) #in-control covariance matrix
t<-1
z[1,]<-rep(0,4)
for(t in 1:1000){
x<-mvrnorm(1000,m,D) #in-control variable
x1[,t]<-c(x[1:4])
u<-1
for (u in 1:4){
        r<-rank(x1[u,1:t])
        R<-r[t]
        ER<-(t+1)/2
        varR<-((t+1)*(t-1))/12
        R<-(R-ER)/sqrt(varR)
R1[u]<-R
u<-u+1
}
R1
x3[t,]<-R1
t<-t+1
}
x4<-x3[1:1000,]
j<-1
for(j in 1:2000){     #repeat 2000 times
i<-1
while (abs(z[i])<0.2508){
i<-i+1
x<-mvrnorm(1,m,D) #in-control variable
x1[,i]<-c(x[1:4])
```

```
u<-1
for (u in 1:4){
        r<-rank(x1[u,1:i])
        R<-r[i]
        ER<-(i+1)/2
        varR<-((i+1)*(i-1))/12
        R<-(R-ER)/sqrt(varR)
R1[u]<-R
u<-u+1
}
R1
x8[i,]<-R1
x9<-x8[1:i,]
x6<-rbind(x4[-1,],x9[-1,])
x7<-cov(x6)
z[i,]<-(t(R1))%*%I+z[i-1,]%*%(I1-I)
H<-(lambda)/(2-lambda)*x7
z1<-z[i,]
p[i]<-z1%*%solve(H)%*%(t(t(z1)))
}
m2[j]<-i}    #run length, RL
mean(m2)    #caculate the in-control average of run length, IC ARL
```
